# Early Chordate Origin of the Vertebrate Integrin αI Domains

**DOI:** 10.1371/journal.pone.0112064

**Published:** 2014-11-19

**Authors:** Bhanupratap Singh Chouhan, Jarmo Käpylä, Konstantin Denessiouk, Alexander Denesyuk, Jyrki Heino, Mark S. Johnson

**Affiliations:** 1 Structural Bioinformatics Laboratory, Biochemistry, Department of Biosciences, Åbo Akademi University, Turku, Finland; 2 Department of Biochemistry, University of Turku, Turku, Finland; Lerner Research Institute, United States of America

## Abstract

Half of the 18 human integrins α subunits have an inserted αI domain yet none have been observed in species that have diverged prior to the appearance of the urochordates (ascidians). The urochordate integrin αI domains are not human orthologues but paralogues, but orthologues of human αI domains extend throughout later-diverging vertebrates and are observed in the bony fish with duplicate isoforms. Here, we report evidence for orthologues of human integrins with αI domains in the agnathostomes (jawless vertebrates) and later diverging species. Sequence comparisons, phylogenetic analyses and molecular modeling show that one nearly full-length sequence from lamprey and two additional fragments include the entire integrin αI domain region, have the hallmarks of collagen-binding integrin αI domains, and we show that the corresponding recombinant proteins recognize the collagen GFOGER motifs in a metal dependent manner, unlike the α1I domain of the ascidian *C. intestinalis*. The presence of a functional collagen receptor integrin αI domain supports the origin of orthologues of the human integrins with αI domains prior to the earliest diverging extant vertebrates, a domain that has been conserved and diversified throughout the vertebrate lineage.

## Introduction

Integrins are multi-domain cell-surface receptors that fulfill numerous function roles at the level of cell-cell communication and interactions between cells and proteins of the extracellular matrix (for a review, see [1]). Integrins have an early origin, preceding the first metazoans [2], with most component domains identifiable in bacterial sequences (see e.g. [3]–[5]; reviewed in [6]) and, despite multicellular species that do not have integrins (e.g. fungi and plants), integrins were likely necessary and greatly facilitated the development and diversification of multicellular animals. The bidirectional signaling mediated by integrins enables changes relative to the external environment when instigated by cytoplasmic events in individual cells or promotes cellular changes as a result of ligand binding to the external ectodomain. Consider, for example, the dynamic processes involved in tissue remodeling and wound repair, where e.g. cells accumulate on collagen fibers of the ECM and cells of the immune system bind at sites of inflammation, but where these cells also will need to detach and relocate.

**Figure 1:**
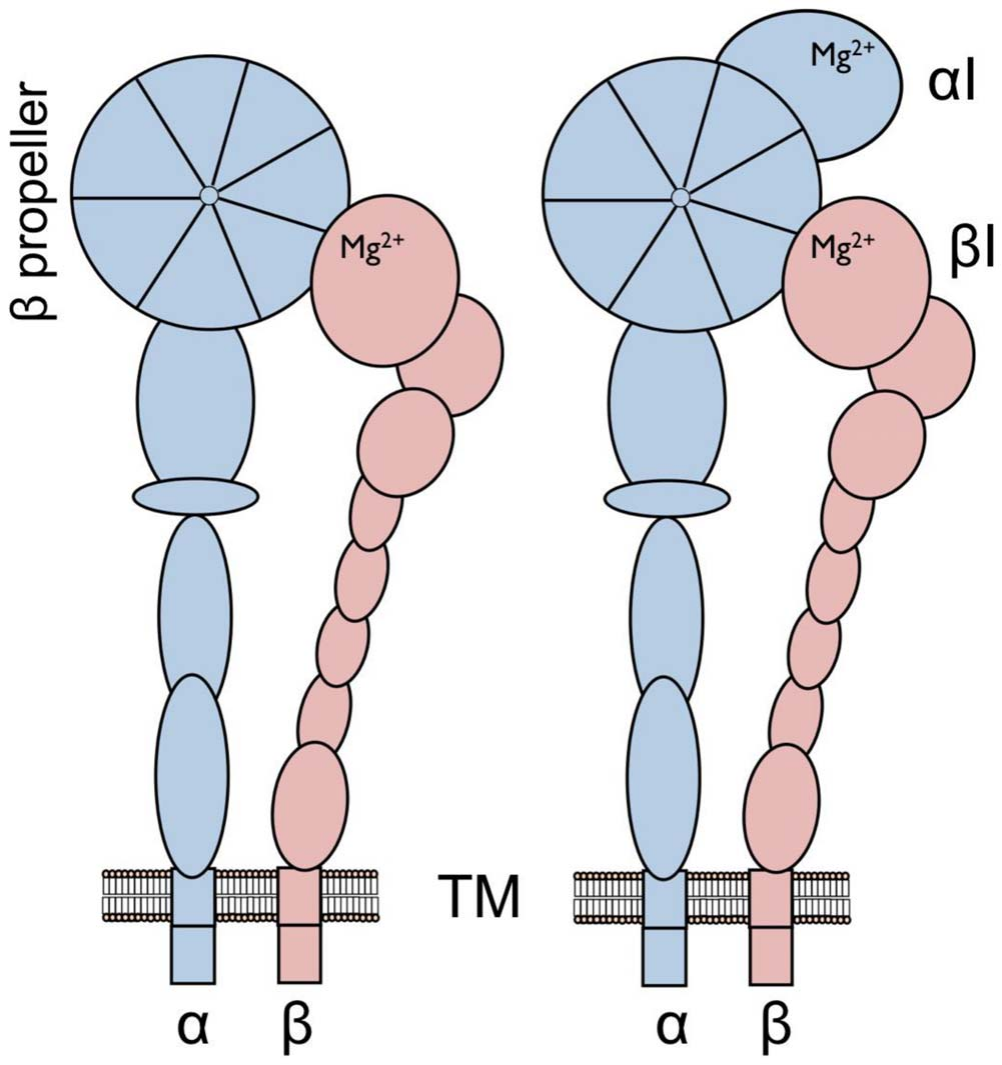
Schematic representation of integrin heterodimers. Integrins are large heterodimeric, bi-directionally signaling, cell surface receptors that consist of a large extracellular ectodomain, a transmembrane region and relatively short intracellular “tails” (right). (A) The constituent α and β subunits are non-covalently associated and the α subunit (ca. 1100 residues) is generally larger than the β subunit (ca. 800 residues). (B) Half of the human integrin α subunits – α1, α2, α10 and α11 of the collagen receptors and αD, αX, αL, αM and αE of the leukocyte clade – contain an additional domain known as the “inserted” αI domain, which buds out between the second and third repeat of the β-propeller domain located at the α subunit N-terminus. The αI domain is a member of the von Willebrand factor A domain family present in many other proteins, including all integrin β subunits and many proteins related to the extracellular matrix, and it is known to adapt the Rossmann fold. The αI domain contains the highly solvent-exposed MIDAS site (Mg^2+^) where natural ligands bind via a negatively-charged amino acid glutamate. The βI-like domain is located towards the N-terminus in β subunits and acts as the recognition site for external ligands in those integrin heterodimers that do not have the αI domain (A), but binds a glutamate residue – an intrinsic ligand – from the αI domain in the collagen receptor and leukocyte clade α subunits (B).

In humans there are 24 integrin heterodimers that have been observed to form from 18 α subunits and 8 β subunits [7]. Half of the α subunits have an extra “inserted” I domain [8] or “A” domain [9] (see Fig. 1). Of the nine integrins with αI domains, five have immune system functions: αLβ2, αMβ2, αDβ2, αXβ2 and αEβ7; and four are collagen receptors: α1β1, α2β1, α10β1 and α11β1. The first X-ray structures of integrins deposited within the Protein Data Bank (PDB; [10]) have focused on the αI domain in human integrin α subunits: e.g. αM (PDB code: 1IDO and 1JLM; [11], [12]) and αL (1LFA; [13]) of the immune system type; and α2 without (PDB code: 1A0X; [14]) and with (1DZI; [15]) collagen-like triple-helical GFOGER peptide bound. In 2010, the αXI domain was solved within the ectodomain context of the αβ subunit complex (3K6S; [16]).

The αI domains are Rossmann folds, but more specifically they belong to the von Willebrand factor type A-like fold (vWA-like, the SCOP database, [17]) and the sequences are categorized to von Willebrand factor type A protein ECM (vWA_ECM) in the NCBI Conserved Domain Database (CDD, [18]). The integrin αI domains (cd01469 sequence cluster; vWA_integrins_α_subunit) are only one of nine domain subfamilies (CDD ID: cd01450, vWFA_subfamily_ECM) that includes at least 110 different eukaryotic domains [6].

**Figure 2:**
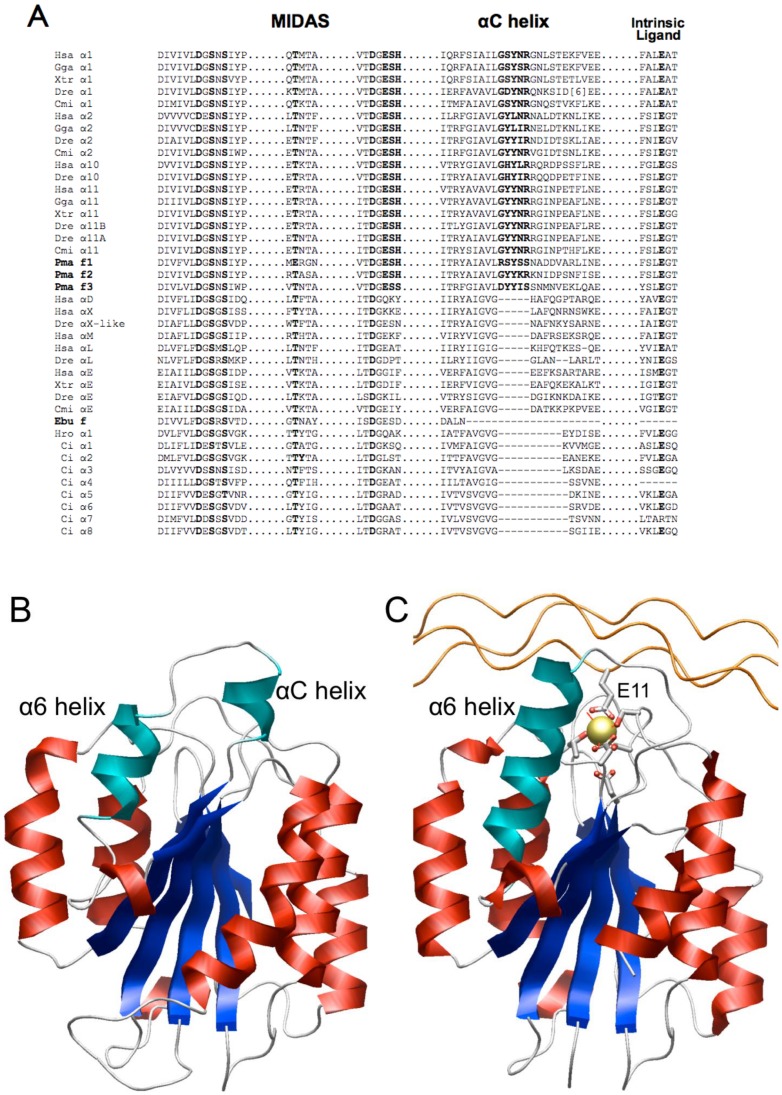
Key features of the integrin αI domain. (A) Alignment of representative sequences, including the three sea lamprey fragments, one short EST fragment derived from the inshore hagfish genome, and four sequences from the elephant shark genome (highlighted in bold). The residues DxSxS…D…T of MIDAS (in bold) function to bind directly or via water molecules to the metal ion where natural ligands bind via a glutamate residue. The sequence ESH (bold) is characteristic of collagen-binding αI domains; the αC helix (bold) is a distinctive hallmark of the collagen receptor α subunits. The intrinsic glutamate ligand (bold) of the αI domain binds to MIDAS of the βI-like domain in integrins that have the inserted αI domain. Structure of the α2I domain without (B) (PDB code: 1AOX; [14]) and with (C) bound GFOGER tripeptide (PDB code: 1DZI; [15]). The peptide binds to the metal (yellow sphere) at MIDAS via glutamate E11 of the peptide. Consequently, the αC helix unravels and the α6 helix lengthens.

All integrin β subunits contain a βI-like domain (Fig. 1) and, for example, in the αVβ3 integrin that does not have an inserted αI domain, protein ligands bind via the RGD sequence motif (and variants; see e.g. [19]) located on external loops where the aspartic acid binds to the metal ion dependent adhesion site (MIDAS) of the βI-like domain and arginine binds to the β-propeller domain of the α subunit (1L5G; [20]). MIDAS in the αI domain is also key to ligand recognition and function of integrins with αI domains as seen in the three-dimensional structures of α2I-GFOGER [15] and α1I-GLOGEN (PDB code: 2M32, [21]) where the glutamate of the triple-helical collagen-like peptides bind at a coordinating position to a divalent metal cation. Similarly, glutamate e.g. from ICAM1 (1MQ8, [22]; 3TCX, [23]), ICAM3 (1T0P, [24]) and ICAM5 (3BN3, [25]) bind to MIDAS of the αLI domain. The collagen binding integrins and those that recognize leukocytes also have recognizable differences, having the αC helix containing a key tyrosine residue (Y285 in the α2I domain; 1AOX) and present only in the collagen receptor αI domains [14] – an easy-to-scan sequence feature observable in alignments (Fig. 2; [26], [27]), observed in the ligand-free structures of the α1I and α2I domains but unraveled (Y285 moves by over 17 Å forming a hydrogen bond with S316; 1DZI) after the conformational changes accompanying ligand binding.

Integrin sequences with αI domains have not been observed in echinoderms [4] nor in the genome [28] of the earliest-diverging chordate – *Branchiostoma floridae*, the lancelet [6], [29], but integrins do make their initial appearance in another early chordate species, with one αI domain sequence identified in the tunicate *Halocynthia roretzi* [30] and eight α subunits with αI domains identified [29], [31] among the genomic sequencing data [32] of *Ciona intestinalis*. Tunicate integrins with αI domains are not orthologues of the nine human integrin α subunits with I domains [4], [6], [29], [31], and none of the tunicate sequences contain the αC helix that characterizes the human collagen receptor integrins (Fig. 2).

The I domain leads to a dramatic alteration to the integrin ligand-recognition structure in that it shifts the ligand recognition site (see Fig. 1) from a narrow space where an exposed loop on the protein ligand that can cross-link MIDAS of the βI-like domain with the β-propeller domain to a more exposed site that recognizes larger, tubular-shaped and bulkier domain ligands, e.g. collagen fibers bundled into large macroscopic structures and immunoglobulin-fold ICAM domains. With the α2I domain, other, opportunistic ligands such as a snake venom metalloproteinase and echovirus 1 [33]–[36] very likely bind to the αI domain, covering the MIDAS site, but not directly via a ligand-metal interaction at MIDAS. In integrins with an αI domain, the βI-like domain of the β subunit assumes a new role, by binding a negatively-charged residue (e.g. E336 in α2I) from the α subunit as an “intrinsic ligand”, helping to stabilize one of several conformations in the dynamic, mechanical responses to bidirectional signaling [16], [36]–[38].

Here, we have sought to clarify the origins of the integrin α subunits having I domains with features characteristic of the human receptors. In searching for integrin sequences throughout the chordates we identified three sequences from lamprey and possibly one from hagfish that have the hallmarks of αI domains. Furthermore, three fragments from a shark genome study [39], seen earlier [26], two of which have the αC helix, are clearly derived from integrins orthologous to human integrins and now, with the genome published, at least four complete α subunits of integrins with I domains are identifiable. Here, we characterize the features of those sequences and their likely structures and place them within the contextual framework for integrin evolution that has unfolded over the past 25 years.

## Results

### Searches Identify Likely αI Domain Sequences in Cartilaginous Fish and Tunicates

Orthologues of the human integrin α subunits with I domains are found in species extending from the bony fish (Osteichthyes) through to the mammals [6], [29], [40]. Thus, we can bracket the appearance of the integrin α subunits with I domains, having features found in the human receptors, to ancestors of species that appeared since the divergence of the tunicate ascidians and before the appearance of the bony fish. Only a few extant representative groups have diverged after the tunicates and before the bony fish, and some genomic data are available for two Agnathostomes (jawless vertebrates) – *Eptatretus burgeri* (inshore hagfish) and *Petromyzon marinus* (sea lamprey) and from cartilaginous fish (Chondrichthes; sharks/rays/skates/chimaera).

**Figure 3:**
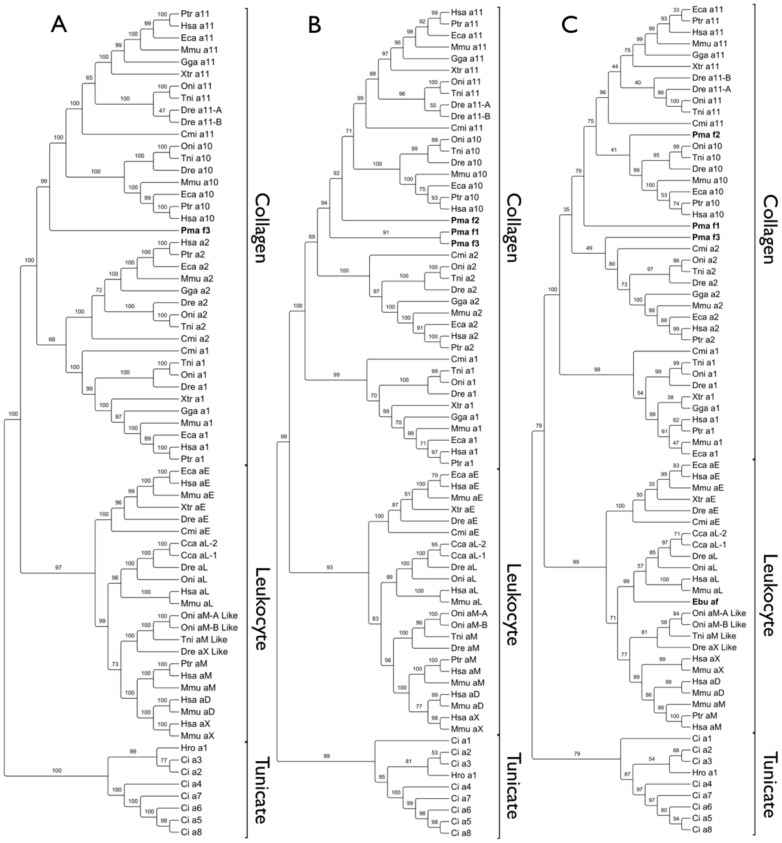
Phylogenetic analysis of integrin sequences with the Maximum Likelihood method. (A) Tree based on the full-length sequence alignment of integrin α subunits derived from the species listed in Table 1. This dataset contains the nearly full-length integrin α subunit from the sea lamprey Pma_f3 (highlighted in bold). (B) Tree based on the aligned common sequence region in all three lamprey sequence fragments Pma_f1, Pma_f2 and Pma_f3 (highlighted in bold). The common region of the α subunit includes three of seven beta propeller repeats (a small portion of repeat number 2, repeat 3 and repeat 4) and the integrin αI domain; the alignment spans about 550 positions. (C) Tree based on the alignment of the integrin αI domain sequences; this dataset includes the three lamprey αI domain sequences Pma_f1, Pma_f2 and Pma_f3 (highlighted in bold) and the hagfish fragment Ebu_f (highlighted in bold). The sequence alignment or the αI domains spans about 250 positions including gaps. Trees were constructed using MEGA by implementing the Whelan and Goldman substitution matrix with frequency model and gamma distribution with invariant sites (WAG+I+G+F). Statistical support for each phylogenetic tree was obtained with 1000 bootstrap replicates and the percentage bootstrap support value is indicated at each node.

We have been regularly searching genomic sequencing data for integrins sequences in order to clarify the origins of different features, especially integrin α subunits, individual domains and αI domains in particular. We conducted searches [26] of all the available genomic assemblies and ESTs from species that diverged after the urochordates and before the bony fish: including *P. marinus*, *E. burgeri*, *Callorhinchus milii*, (chimaera; elephant shark; Australian ghost shark), *Raja erinacea* (little skate) and *Squalus acanthias* (dogfish shark). Although our intuition is that orthologues of human αI domains should be found in cartilaginous fish, our searches of the chimaera, skate and shark assemblies only yielded three short fragments. Two sequence fragments from *C. milii* were very similar to portions of the human integrin I domains α1 (AAVX01128089.1; 55 residues; 76% identical) and α2 (AAVX01352230.1; 55 residues; 71% identical), beginning by matching the αI domain αC helix; a third fragment from *C. milii* matched repeat 5 of the β-propeller domain of human α2 (AAVX01625876.1; 52 residues; 63% identical). Now, with the publication of the genome sequence of *C. milii* [39], there are at least four orthologues of the corresponding human integrin subunits: collagen-binding α1, α2 and α11, and αE from the leukocyte clade (fig. 3).

Searches [26] also identified three sequence fragments from the sea lamprey genome [41]. With more recent updates these fragments include Pma_f1 having two splice variants (ENSPMAP00000003339, 617 amino acids; ENSPMAP00000003342, 582 amino acids), Pma_f2 (ENSPMAP00000008300, 478 amino acids) and Pma_f3 (ENSPMAP00000003839, 1099 amino acids), which is nearly full-length and missing about 120 residues (compared to the α10I and α11I domains) corresponding to the first two repeats from the N-terminus of the β-propeller domain [26]. In this study we have considered the sequence of the larger 617 amino acid splice variant of Pma_f1.

Additionally, one short 133-residue fragment (Ebu_f) of a possible αI domain from the hagfish *E. burgeri* genome [42] was identified by Blast searches (NCBI service) using human αI domain sequences as the query. When compared with the nine human integrin αI domains, the sequences derived from the sea lamprey genome were found to contain the signature αC helix located towards the C-terminal region of the αI domain – the hallmark of the collagen-receptor integrin subunits (Fig. 2). The αC helix region is not found in either the immune system I domains nor in the sequences of the nine tunicate integrin αI domains. The short fragment that is derived from the hagfish terminates just prior to the αC helix (Fig. 1) but sequence searches suggested it may be most similar to a leukocyte clade member, the integrin αLI domain.

### Agnathostome αI Domain Sequences Cluster with Human αI Domains

Here, we have constructed three separate sets of phylogenetic trees from sequence alignments and based on three different tree reconstruction methods. The sequences include representatives from 15 chordate species containing the αI domain (Table 1 and Table S1 in File S1.doc). In addition to sequences from nine human α subunits, sequences are included from other mammals, chicken, a frog (*Xenopus laevis*), four bony fish species (*Tetraodon nigroviridis*, green spotted pufferfish; *Oreochromis niloticus*, Nile tilapia; *Danio rerio*, zebrafish; *Cyprinus carpio*, common carp), four sequences from the elephant shark *C. milii* and sequences from the tunicates *H. roretzi* and *C. intestinalis*. The sets of trees differ in having (a) 69 sequences including the near full-length lamprey Pma_f3 sequence; (b) 72 partial sequences that include the maximum common regions – 406 to 409 residues shared by the 3 lamprey sequences Pma_f1-3; and (c) 73 αI domain regions of approximately 200 residues that include the hagfish sequence fragment Ebu_f and Pma_f1-3. The 701-residue sequence fragment of α1 from *C. intestinalis* is included in the αI domain and common regions trees.

**Table 1:** Chordate genomes and EST assemblies utilized for the integrin phylogenetic analysis.

Organism	Sequence code used	Scientific name	Subphylum/Superclass/Class/Subclass/Order
Human	Hsa	*Homo sapiens*	Vertebrata/Tetrapoda/Mammalia/Theria/Primates
Chimpanzee	Ptr	*Pan troglodytes*	Vertebrata/Tetrapoda/Mammalia/Theria/Primates
Horse	Eca	*Equus caballus*	Vertebrata/Tetrapoda/Mammalia/Theria/Perissodactyla
Mouse	Mmu	*Mus musculus*	Vertebrata/Tetrapoda/Mammalia/Theria/Rodentia
Chicken	Gga	*Gallus gallus*	Vertebrata/Tetrapoda/Aves/-/Galliformes
African clawed frog	Xtr	*Xenopus laevis*	Vertebrata/Tetrapoda/Amphibia/-/Anura
Green spotted pufferfish	Tni	*Tetraodon nigroviridis*	Vertebrata/Osteichthyes/Actinopterygii/Neopterygii/Tetraodontiformes
Nile tilapia	Oni	*Oreochromis niloticus*	Vertebrata/Osteichthyes/Actinopterygii/Neopterygii/Perciformes
Zebrafish	Dre	*Danio rerio*	Vertebrata/Osteichthyes/Actinopterygii/Neopterygii/Cypriniformes
Common carp	Cca	*Cyprinus carpio*	Vertebrata/Osteichthyes/Actinopterygii/Neopterygii/Cypriniformes
Elephant shark	Cmi	*Callorhinchus milii*	Vertebrata/Chondrichthyes/Chondrichthyes/Holocephali/Chimaeriformes
Inshore hagfish	Ebu	*Eptatretus burgeri*	Vertebrata/-/Myxini/-/Myxiniformes
Sea lamprey	Pma	*Petromyzon marinus*	Vertebrata/-/Cephalaspidomorphi/-/Petromyzontiformes
Vase tunicate	Ci	*Ciona intestinalis*	Tunicata/-/Ascidiacea/-/Enterogona
Sea pineapple	Hro	*Halocynthia roretzi*	Tunicata/-/Ascidiacea/-/Pleurogona

“-” indicates that the classification is not available.

**Figure 4:**
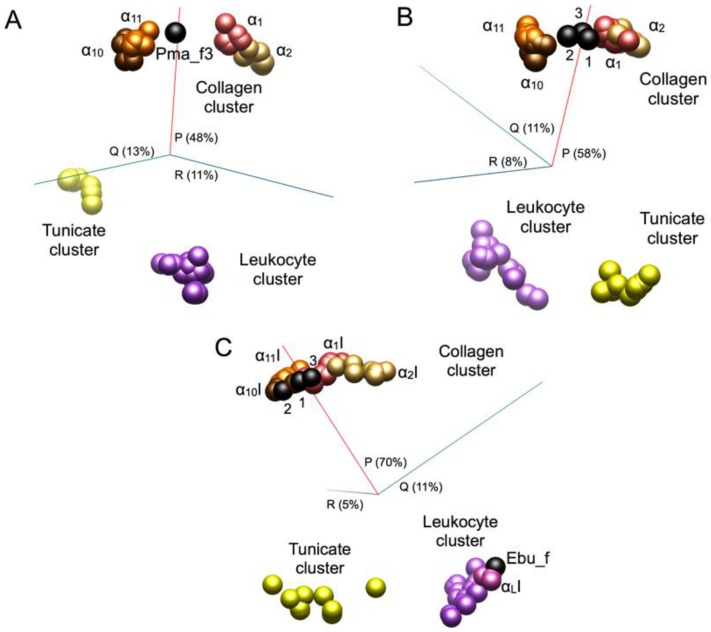
Multivariate plots reflect the details of the phylogenetic analyses. (A) Full-length sequences of the integrin α subunit, (B) sequence regions shared in common with Pma_f1-3, and (C) the αI domain region. The plots were based on distances (JTT scoring) obtained from the sequence alignments. The plots show the relationships among the sequences for the three most informative dimensions and the percentage variance accounted for along the axis is indicated.

Phylogenetic trees were inferred from sequence pairwise distances (using either the JTT distance matrix [43]; or the Whelan and Goldman (WAG) matrix [44]) obtained from the aligned sequences and based on implementations of the Maximum Likelihood (ML; Fig. 3), Bayesian (Fig. S1 in File S1.doc) and Neighbor Joining (NJ; Fig. S2 in File S1.doc) methods as described in the Materials and Methods. Multivariate 3D plots were prepared based on the JTT distance data and lend support to the tree topologies (Fig. 4).

The clusterings represented by trees constructed using the ML (Fig. 3) and Bayesian (Fig. S1 in File S1.doc) methods reflect the identical segregation of major groups and most details within the groups also match, and are in agreement with published trees ([4], [6], [29], [31], [40], [45]–[51] among others). In general, the tunicate sequences cluster as a single clade and as an apparent outlier to the remaining integrin I domains. The vertebrate integrin sequences segregate into two major clusters corresponding to the immune system or leukocyte clade integrins and those of the collagen receptors, and both clusters subdivide according to the generally accepted subgroups (Fig. 3A). Fish sequences exhibit subtype pairs (e.g. zebrafish α11A and α11B) and the fish cluster branching after the αE and αL branches appear to have diverged prior to the αM-αD-αX diversification found in mammals. Some discrepancies do appear, e.g. α1/α2 subunit clustering in the NJ tree (Fig. S2A in File S1.doc; also indicated by poor bootstrap replication) and when trees are based on the shorter, less-informative sequence fragments i.e. the αI domain region. The fragments from the elephant shark *C. milii* that were observed by us earlier clearly corresponded to orthologues of the human α1I and α2I domains. Three full-length sequences now available from the published genome sequence cluster appropriately as outliers to the α1, α2 and α10 collagen receptor integrins, prior to the bony fish representatives, consistent with them being true orthologues of these vertebrate integrins. Similarly, the αE sequence of *C. milii* appears to be a true orthologue since it also branches just prior to the zebrafish sequence in the αE cluster. Thus, it appears that true orthologues of at least four integrins with αI domains, from both collagen receptors and from the immune system integrins, found in species from bony fish to human are also present in the cartilaginous fish.

The ML tree based on the largest common fragment from the three lamprey sequences (Fig. 3A) places the lamprey Pma_f3 sequence after the α1/α2 divergence and as an outlier of the α10/α11 clade, in agreement with the Bayesian (Fig. S1A in File S1.doc) and NJ (Fig. S2A in File S1.doc) trees. The bootstrap reproducibility of the ML and NJ trees are near 100% (1000 replicates) for nodes where Pma_f3 branches. The posterior probabilities assigned to the branches in the Bayesian tree are 100% for most branches and for the node leading to the Pma_f3 branch.

Similarly, the ML, Bayesian and NJ trees (Fig. 3B, Figs. S1B and S2B in File S1.doc) based on the largest common region shared by all three lamprey sequences places the three lamprey sequences as an outlier of the α10/α11 clade, where Pma_f1 and Pma_f3 cluster together and adjacent to Pma_f2. There is clearly more noise in the trees overall, reflected in differences within the branch orders among the trees and with the full-length trees, and less reliable bootstrap and probability indicators at nearby nodes.

Although the alignments of the sequences corresponding to the shorter αI domain regions are very reliable, the similarity differences over the αI domain are less discriminating than those from the longer sequences. The trees based only on the αI domain regions (Fig. 3C, and Figs. S1C and S2C in File S1.doc) reflect the general features of the other trees based on the longer sequences, but the level of noise is even higher and there are more discrepancies, e.g. in the collagen integrin subdivisions. Nonetheless, the lamprey sequences cluster with the collagen receptor αI domains, although their locations are more variable compared to the full-length and common-segment trees, but then the support for the trees in the vicinity of the lamprey sequences is also poor. The features of all three sets of trees are also reflected in the multivariate plots (Fig. 4).

The hagfish fragment (Ebu_f) ends prior to the αC helical region (Fig. 2). A search of the fragment using the Blast server ([52]; blast.ncbi.nlm.nih.gov) identifies as the closest matches multiple αL integrins, and in all three trees (Fig. 3C, and Figs. S1C and S2C in File S1.doc) the sequence branches off with the immune cell receptor αLI domains, and this is consistent with the multivariate analysis of the distance data (Fig. 4C). Thus, the short fragment from the hagfish (Ebu_f) may be a homologue of the leukocyte specific integrin α subunit, but one must be cautious given the short fragment and lack of other clear distinguishing features in the sequence.

### Functional Residues are Shared between Human and Lamprey αI Domains

Key residues involved in αI domain recognition of the collagen-like GFOGER and GLOGEN tripeptides were identified from known representative three-dimensional structures of complexes using Surf2 (MS Johnson, unpublished), and then we examined the similarities and differences among equivalent residues in the human set of integrin αI domains and the residues present in the agnathostome sequence fragments (Table 2, and Tables S2 and S3 in File S1.doc).

**Table 2:** Residues in the α2I domain structure within 4.2 Å (non-hydrogen atoms) of the bound GFOGER tripeptide and equivalent residues in the other human and lamprey αI domains, and the fragment from the hagfish.

α2I, 1DZI, 2.10 Å	*S153*	N154	*S155*	Y157	N189	Q215	G217	G218	D219	L220	*T221*	E256	S257	H258	Y285	L286
α_1_I, 1PT6, 1.87 Å	S152	N153	S154	Y156	E188	Q214	G216	G217	R218	Q219	T220	E255	S256	H257	S284	Y285
α_10_I	S	N	S	Y	E	R	E	G	R	E	T	E	S	H	H	Y
α_11_I	S	N	S	Y	E	Q	G	G	T	E	T	E	S	H	Y	Y
Pma_f1	S	N	S	Y	A	R	W	G	M	E	R†	E	S	H	S	Y
Pma_f2	S	N	S	Y	F	S	P	F	V	R	T	E	S	H	Y	Y
Pma_f3	S	N	S	W	E	Q	G	G	K	V	T	E	S	S	Y	Y
Ebu_af	S	R	S	T	S	Q	K	A	*	G	T	E	S	D	?	?
α_L_I, 3F74, 1.70 Å	S139	M140	S141	Q143	T175	H201	L203	L204	*	L205	T206	E241	A242	T243	*	*
α_M_I, 1IDO, 1.70 Å	S141	G142	S143	I146	E178	Q204	L206	G207	*	R208	T209	E244	K245	F246	*	*
α_X_I, 1N3Y, 1.65 Å	S140	G141	S142	S144	N176	Q202	Q204	G205	*	F206	T207	K242	K243	E244	*	*
α_D_I	S	G	S	D	N	Q	K	G	*	L	T	Q	K	Y	*	*
α_E_I	S	G	S	D	G	Q	G	S	*	V	T	G	I	F	*	*

Where available, the sequence numbering is from a three-dimensional structure (PDB codes and resolution are indicated). The metal ion at MIDAS is covalently bound to the tripeptide ligand. Residues from MIDAS (S153, S155 and T221 in α2I, 1DZI) are in italics and two residues, D151 and D254 in α2I (not listed), are absolutely conserved across all of the sequences and bind to the metal at MIDAS via a water molecule (WAT2001).

*, no equivalent or aligned residue; ?, residue not present in the sequence fragment;

† alignment uncertain at the position - no threonine present nearby in the sequence and replacement of arginine with threonine did not alter binding to collagens of the expressed mutant (data not shown).

**Figure 5:**
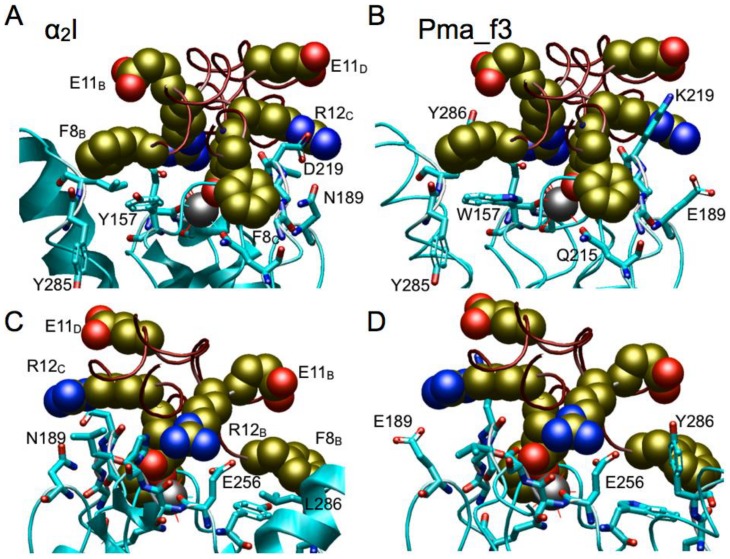
Views of (A, C) the structure of the α2I domain with bound GFOGER tripeptide (PDB code: 1DZI) and (B, D) a model constructed for lamprey Pma_f3 αI domain; (C) and (D) are rotated approximately 180° from the view in (A) and (B). The model of Pma_f3 αI was superposed on the α2I-peptide complex in order to place the peptide in the same relative position in the model of Pma_f3. Relevant residue side chains of the peptide are shown as CPK models and residues from the αI domains are shown as ball and stick models. For clarity, residues and water molecules binding the metal (grey sphere) at MIDAS are not shown.

The integrin αI domain provides a highly-exposed surface for ligand recognition. The central metal is presumably Mg^2+^ at the MIDAS site and binds glutamic acid of ligands, although Co^2+^ used in the crystallization is present in the α2I domain and binds E11 from one chain of the GFOGER tripeptide ligand in the complex structure (Fig. 5; [15]). Similarly, a glutamate of the GLOGEN tripeptide binds to the metal ion at MIDAS in the α1I domain structure of the complex [21] but the peptide is rotated about the glutamate with respect to the α2I-GFOGER tripeptide complex structure, which may suggest that different collagen recognition sequences bind at different rotational positions on the surface of a particular collagen-binding αI domain. In the leukocyte clade αLI domain structures with bound ICAM-1 D1 (3TCX; [23]), ICAM-3 (1T0P; [24]) and ICAM-5 (3BN3; [25]), immunoglobulin-like fold domains bind to αLI respectively via E34, E37 and E37 to the metal at MIDAS.

The residues from the human α2I domain within 4.2 Å of the tripeptide are shown in Table 2 along with the equivalent sequences in the other eight human αI domains, the three lamprey sequences and the hagfish fragment. Similarly, the nearby residues in the α1I-GLOGEN [21] and αLI-ICAM3 [24] complexes are compared with the other sequences (Tables S2 and S3 in File S1.doc). Residues of MIDAS are absolutely conserved with the exception of Pma_f1, where there is no nearby equivalent residue to T221 in the α2I domain. Glutamate in the sequence “MER” in Pma_f1 may be able to fulfill that role in binding metal, but this is solely based on modeling of the structure and has not yet been tested experimentally. There are clear differences with the leukocyte αI domains as well as similarities. D219 and equivalent residues in collagen-binding αI domains are important for collagen selectivity [53], where residue swaps at this position, e.g. D219R in α2I and R218D in α1I, exchange the collagen preferences of α2I (the wild type prefers collagen I-III) and α1I/α10I (prefer collagen types IV and VI). This position is absent – a gap – in the leukocyte sequences and in the sequence of Ebu_f. Two residues from the αC helix, Y285 and L286, have equivalent residues in the collagen receptor αI domains and Pma_f1-3, but they are absent in the leukocyte domains; the Ebu_f sequence fragment ends prior to this region.

Residues from the lamprey sequences clearly look most similar to the collagen receptor αI domain residues involved in binding than to the corresponding residues of the leukocyte clade (Table 2). The similarity is reiterated in the corresponding analysis made for α1I-GLOGEN interactions (Table S2 in File S1.doc) and αLI-ICAM3 interactions (Table S3 in File S1.doc), suggesting that the lamprey sequences should recognize multiple collagen subtypes just as the human collagen receptor αI domains do. The sequence ESH (also see Fig. 2) in α2I domain surrounds R12_B_ in the GFOGER peptide complex and H118 from α1I domain forms a key interaction with N213_C_ of the GLOGEN tripeptide in the complex; this sequence is conserved in Pma_f1 and Pma_f2, and ESS in Pma_f3, and ESD in Ebu_f, but less conserved in the leukocyte αI domains (Table 2).

In order to evaluate the potential of the lamprey αI domain sequences for binding collagen, structures were modeled for the three lamprey αI domains with GFOGER triple-helical peptide based on the α2I complex structure (1DZI; [15]) and a wider set of known X-ray structures of αI domains was used to optimize the alignments for structure modeling.

Structural models were built for the lamprey sequences and a comparison of the key features of the X-ray structure of the α2I-GFOGER complex (Fig. 5A and C) and the structural model built for Pma_f3 (Fig. 5B and D) show extensive similarities. Pma_f3 is overall 44% identical with the α2I domain sequence and only one two-position deletion is present in Pma_f3, mapping to the opposite end of the αI domain from MIDAS. Of 18 residues from α2I domain, 16 within 4.2 Å of GFOGER and two other residues that are part of the MIDAS motif, 12 of 18 residues are identical in Pma_f3 (Fig. 2 and Table 2) and, correspondingly, 14 of 18 residues are identical between Pma_f3 and the α11I domain. This includes all five metal-binding residues at MIDAS (i.e. D151, S153, S155, T221 and D254) – all are fully conserved in αI domains, even in the tunicates (Fig. 2) and in some other non-integrin proteins with vWFA domains. Two of three residues important for binding R12_B_ of the GFOGER tripeptide to α2I are also conserved and the replacement of serine for histidine in Pma_f3 would also support interactions with arginine R12_B_ of the peptide. In the model constructed for Pma_f3 (fig. 5B and D), the sequence features at the ligand binding site in the vicinity of where R12_C_ binds to α2I are unique, as it is for the other αI domains, but many features are seen in common with one or more of the human collagen-binding αI domains. In the human collagen receptors, the residue at the position equivalent to D219 in the α2I domain (R218 in α1I) largely determines collagen subtype preferences [53]. This residue is lysine (K219) in Pma_f3 and could reach E11D and form a strong electrostatic interaction that is seen in models for both human α1I and α10I domains where arginine is present. As positioned in the model, E189 in Pma_f3 would interact strongly with R12_C_ of the peptide and this residue is also present in α1I, α10I and α11I.

Pma_f2, like Pma_f1, is identical in sequence at 9 of 16 ligand-interacting positions seen for the α2I domain. One key position in α2I, T221, functions to chelate the metal ion at MIDAS and the equivalent residue in the Pma_f1 sequence is uncertain and there is no threonine residue nearby. In Table 2, the alignment of the Pma_f1 sequence ^219^MER^221^ with ^219^DLT^221^ in the α2I domain cannot be correct as the large arginine side chain in the Pma_f1 sequence cannot substitute for threonine (the engineered, expressed R221T mutant behaves like the expressed wild-type Pma_f1 αI domain; data not shown) but it may be that the adjacent E220 can substitute for threonine; it remains to be tested.

### Sea Lamprey αI Domains Recognize Different Mammalian Collagen Types and GFOGER tripeptide

**Figure 6:**
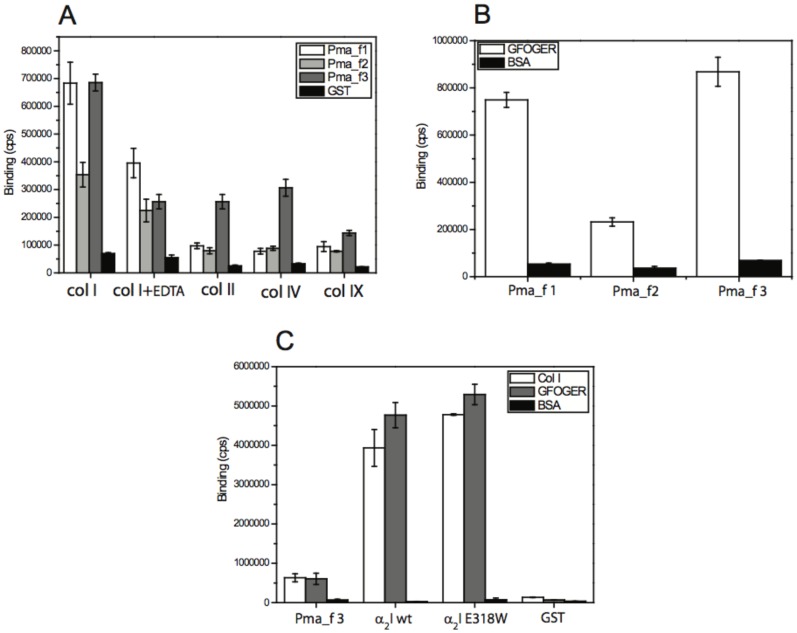
Lamprey αI domains recognize mammalian collagens and the GFOGER-motif in a metal-dependent manner. (A) Binding of Pma_f αI domains to various mammalian collagens at a fixed (400 nM) concentration. The EDTA concentration was 10 mM. GST binding serves as a negative control. (B) Binding of Pma_f αI domains to triple-helical GFOGER-tripeptide at a fixed concentration (400 nM). Binding to BSA serves as a negative control. (C) Binding of Pma_f3 αI, human α2I wt, and human α2I E318W domains to rat collagen I, GFOGER-peptide, and BSA. Binding of GST serves as a control.

The three sea lamprey αI domain sequences of Pma_f1, Pma_f2, and Pma_f3 were synthesized and cloned into expression vectors pGEX-2T producing the recombinant GST-fusion proteins. Recombinant proteins were expressed in the *E. coli* strain BL21 tuner. The expressed proteins were sufficiently pure for kinetic experiments to be carried out. A minor amount of GST was observed in each protein preparation and in Pma_f3 preparations a small amount of processed fusion protein was occasionally observed (Fig. S3 in File S1.doc). The ability of recombinant Pma αI domains to recognize and bind to various collagens was tested with a solid-phase assay as described previously [54]. Binding studies, performed using a fixed concentration of Pma αI domain (400 nM), showed that all recombinant Pma αI domains recognize and bind to several different collagens types: rat collagen I and bovine collagen II (fibrillar collagens), mouse collagen IV (network-forming collagen), and recombinant human collagen IX (FACIT) (Fig. 6A). The highest binding for all Pma αI domains is seen with rat collagen I and generally Pma_f3 αI showed the highest binding with all ligands tested. All Pma αI domains show metal-dependence in binding rat collagen I since when recombinant Pma αI domains were incubated with EDTA in the binding step the observed binding levels were clearly lower (Fig. 6A).

GFOGER is a well-known motif in collagen receptor integrins [55] and one of the most important recognition sequences in, e.g. collagen I. We tested whether triple-helical GFOGER peptide could be recognized by recombinant Pma αI domains. All Pma αI domains bind the GFOGER peptide (Fig. 6B), showing a similar binding profile to the rat collagen I binding profile (Fig. 6A); Pma_f1 and Pma_f3 αI domains show the highest binding and the Pma_f2 αI domain binds to a lesser extent.

In order to compare the binding of Pma αI domains and human collagen receptor integrin αI domains, Pma_f3 αI domain, human wild type α2I wt and human α2I E318W (“open conformation” mutant) were tested for binding to rat collagen I. Recombinant Pma_f3 αI domain shows significantly lower binding levels at a high αI concentration (400 nM) (Fig. 6C), possibly indicating that there is a lower number of binding sites available on rat collagen I for Pma_f3 αI domain than for human α2I wt or human α2I E318W. It is known that for human α2I wt there are at least three high-affinity binding sites on bovine collagen I [56] and a few sites with lower affinity [57].

### Pma_f1 and Pma_f3 αI Domains Bind Rat Collagen I at Relatively High Affinity

**Figure 7:**
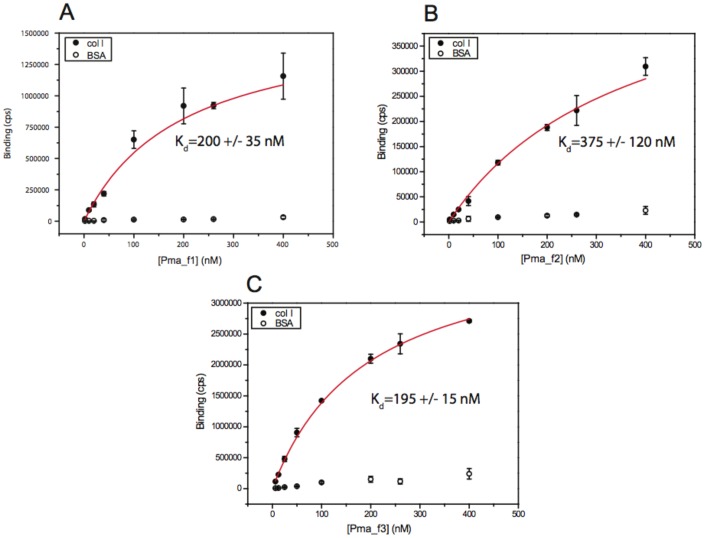
Binding of Pma_f αI domains to rat collagen I as a function of the concentration of Pma_f αI. (A–C) Binding affinities of Pma_f αI domains to rat collagen I were estimated by fitting binding data using a hyperbolic function, which is identical to Hill's equation when h = 1. BSA was used as a control.

In order to determine the binding affinity of recombinant Pma αI domains we tested their binding to rat collagen I at various αI domain concentrations and estimated the affinity as described previously [54], [58]–[59] (Fig. 7). Recombinant αI domains of Pma_f1 and Pma_f3 show clear saturation at higher αI domain concentrations and estimates for the apparent affinity constants can be made (the Kd for Pma_f1 αI is 200±35 nM and the Kd for Pma_f3 αI is 195±15 nM). Recombinant Pma_f2 αI does not indicate clear saturation, which leads to a poorer estimate of the Kd (375±120 nM). The Kd values for lamprey Pma_f1 αI and Pma_f3 αI are comparable to the affinities we have measured typically for the binding of human α2I wt to mouse collagen IV [53].

## Discussion

The basic integrin heterodimeric structure arose early (Fig. 1A), probably within a single-cell eukaryote [2], thus predating the first metazoans. The integrin was key for recognizing important extracellular matrix proteins e.g. fibronectin, having roles in, for example, cell adhesion, cell migration and tissue remodeling. Ligands with short e.g. RGD and LVD recognition sequences form a direct interaction at the βI-like domain of the β subunit via aspartate with the metal cation at MIDAS, and arginine in RGD cross-links via salt bridges with an aspartate residue in the β-propeller domain of the α subunit (see e.g. the X-ray structure of the αVβ3 ectodomain with bound RGD peptide; PDB code: 1L5G, [60]). Because of the narrow confines at the subunit-subunit interface (in αVβ3 the distance from ligand atom OD1 of aspartate D5003, bound to Mn^2+^, to the ligand atom NH2 of arginine R5001 is 14.3 Å and the two “walls” of the α subunit, 8.8-9.7 Å between atoms near the aspartate where R5001 binds, restricts the ligand to be an extended chain), the early integrins were limited to the recognition of exposed loop regions of ligands that could occupy the restricted binding cleft and having restricted options for motif specificity. This integrin organization usefully served for the recognition of proteins from the extracellular matrix and cell surfaces with exposed loops but would have been unable to accommodate other, more bulky ligands.

**Figure 8:**
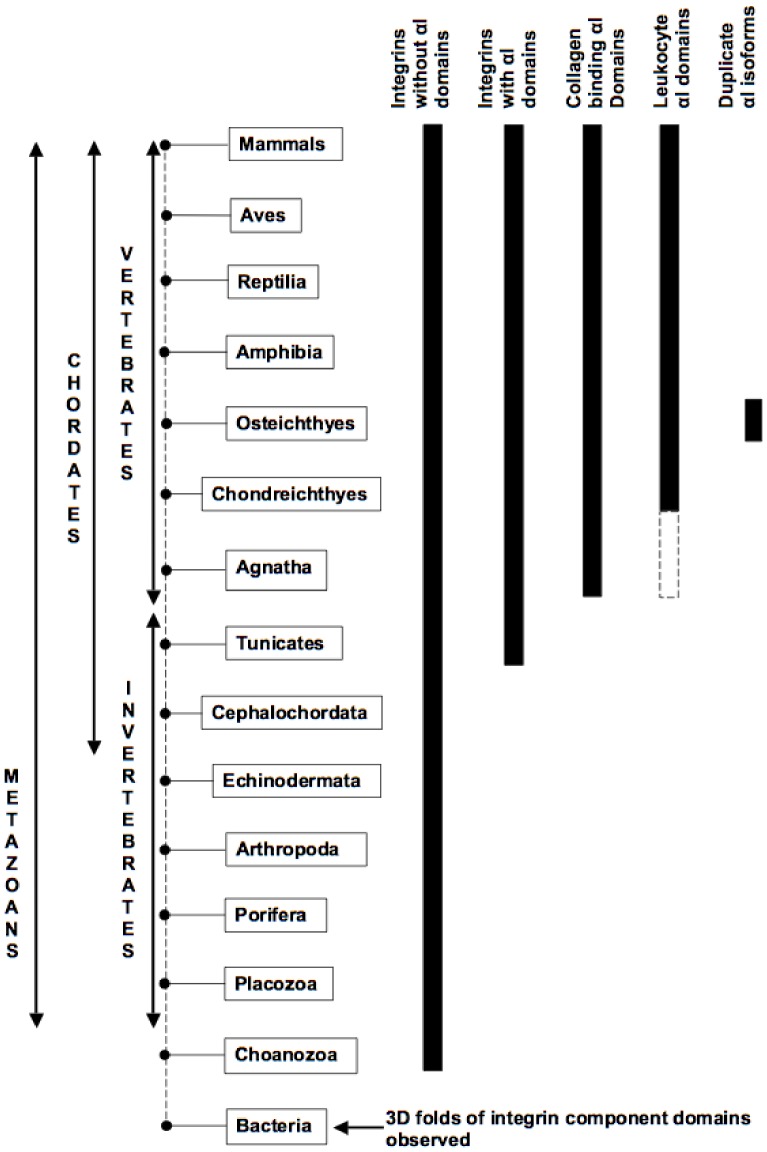
Summary of integrin evolution across a broad range of species: αI domain specialization, as seen in humans, is a vertebrate invention. Individual domains having the same fold class as integrin component domains (i.e. β propeller, immunoglobulin fold, epidermal growth factor fold, vWFA) are observed already in prokaryotes but the earliest diverging sets of identifiable integrin subunits have been observed in the choanozoan *C. owczarzaki*, a single-cell eukaryote. The number of α and β subunits expands with increasing organismal complexity with 18 α and 8 β subunits forming up to 24 heterodimers in humans. Integrins undergo considerable functional diversification with the introduction of the αI domain in some α subunits. Tunicates like *C. intestinalis* and *H. roretzi* are the earliest diverging organisms where integrins with αI domains have been identified, but they are not direct vertebrate orthologues as they form a distinct clade. αI domain containing fragments can be detected in the lamprey *P. marinus* and possibly the hagfish *E. burgeri*; both are extant representatives of the first vertebrates. The lamprey fragments share characteristic features in common with the human collagen-binding αI domain group and they bind different mammalian collagens at MIDAS; four shark sequences are orthologues of the corresponding human α subunits, three collagen binding and one from the leukocyte clade, and duplicate isoforms are observed in observed in bony fish e.g. *D. rerio*, *C. carpio* and *O. niloticus*.

This original organization of the integrin heterodimer is found across the span of metazoan species and is the sole integrin type identified in species diverging prior to the tunicates (Fig. 8). Thus the plan of the α subunit has remained remarkably constant since its inception and half of the integrin α subunits encoded in the human genome abide by this original domain organization.

The insertion of the αI domain into an α subunit occurred approximately 550 MYA, after the deuterostomes first appeared and after the chordate line was established. The αI domain is observed in integrin α subunits from the tunicates but not in the lancelet (Cephalochordata), which is congruent with the lancelet now being acknowledged on the basis of genome comparison studies [61], [28] as having diverged before the ascidians as the earliest extant chordate instead of vice versa as previously thought on the basis of phenotypic characteristics. The αI domain bestowed additional flexibility in terms of ligand recognition by integrins, helping to meet the challenges of major cellular and system-wide changes occurring within the chordate lineage.

The αI domain has a highly solvent-exposed ligand binding surface capable of recognizing larger ligands and surfaces, thus the integrin binding site would no longer be limited to external loops that could access the fairly narrow cleft between the β-propeller and βI-like domains. With the αI domain, ligands bind to the metal at MIDAS via a glutamate residue instead of aspartate found in ligands targeting MIDAS of the βI-like domain. The αI domain allows unfettered access to the binding site facilitating recognition of ICAM immunoglobulin-fold domain surfaces and collagen triple helices bundled into large structures could be more easily accessed and recognized. The more exposed binding site also means that the interaction of the αI domain with ligands involves more residues, upwards of 15 residues in collagen-like peptide and ICAM immunoglobulin fold recognition. As a consequence of the relocation of the binding site, a C-terminal glutamate residue of the αI domain acts as an intrinsic ligand binding to MIDAS of the βI-like domain, participating in the dynamic conformational mechanisms associated with the function of integrins with αI domains.

Here, we show that the first appearance of features characteristic of the collagen receptor integrins, and possibly immune system integrins, are found in the agnathastomes, whereas the integrins with αI domains of the earlier diverging tunicates cluster together and have clearly not specialized into the types observed in humans (Fig. 8). This is not to say that the tunicate αI domains cannot bind collagens or have roles in immune function – Miyazawa et al. [30] have reported that *H. roretzi* α1I functions in a primitive form of complement recognition and Tulla et al. [62] have shown that the *C. intestinalis* α1I domain can bind human recombinant collagen IX that is both metal and MIDAS independent. Orthologues of the human collagen receptor αI domains always have the αC helix and this is found in all three reported lamprey sequences, one of which is a fairly complete α subunit, lacking only the first two repeats of the β-propeller domain. The expressed lamprey αI domains bind mammalian collagens, as shown here but binding, in contrast to *C. intestinalis* α1I, is metal and MIDAS dependent as is the case for mammalian collagen binding with the human αI domains; thus the mechanism of mammalian collagen binding in the tunicate is clearly different from that shared by lamprey and humans. Furthermore, this study shows that the determinants for collagen recognition by integrins with αI domains was established early on in chordate evolution and persists throughout the vertebrates.

I domains in the integrin α subunit have provided a means to diversify chordate integrins to fulfill new tasks associated with the increasing complexity of organs and systems within the chordates, including both complement-based and an adaptive immune system, a circulatory system with the blood clotting, a complex nervous system, cartilaginous and skeletal framework and support system for larger organisms. This study fills in several gaps in our understanding of the evolution of the integrin αI domains, establishing that orthologues of the human integrins with I domains are observed in the agnathostomes, lamprey and perhaps hagfish, present in sharks, but have not been observed in earlier diverging extant chordates or in other invertebrates. The presence of collagen receptor α1, α2 and α11 integrin subunits strongly suggests that integrin α10 must also be present in the cartilaginous fish. The immune system integrins with αI domains appear to diversify fully at a later date than the collagen-recognizing integrins, since only an αE subunit is so far identifiable in the shark *C. milii*. Both αE and αL are present in bony fish but the presence of other bony fish integrins within the leukocyte clade show that the αM/αD/αX specialization had not yet occurred (Fig. 3). It remains uncertain as to the functions of the individual αI integrins in the ascidian *C. intestinalis*, but the function of the integrin fragments with αI domains from the sea lamprey appears clear – they do bind collagens.

## Conclusions

The origin and evolution of integrins with inserted I domains in the α subunit has been clarified by the identification of sea lamprey sequences and their comparison with other chordate integrins. Orthologues of human collagen and some leukocyte receptor integrins extend from the cartilaginous fish, being present in the genome of the elephant shark. The lamprey fragments do not cluster with the earlier diverging tunicates. Instead the fragments share key sequence and thus structural similarities of the collagen receptor integrin clade. Moreover, the expressed lamprey sequences recognize different mammalian collagens at MIDAS as do human collagen receptor integrins and the binding is metal dependent unlike that observed for the tunicate *C. intestinalis* α1I. Leukocyte α subunits are present in cartilaginous fish, possibly in the ascidians too, but they do not diversify into the complete set of five subunits see in humans until after the divergence of the bony fish. Thus, integrin α subunits with inserted I domains whose functions are vertebrate specific were established between the divergence of the ascidians and the appearance of the jawless vertebrates.

## Materials and Methods

### Sequence Searches and Homologue Detection

Searches were made with sequences of human integrin I domain containing α subunits utilizing the BLAST [52] services at the NCBI homepage (http://blast.ncbi.nlm.nih.gov/Blast.cgi) in order to identify potential candidate sequences for this study. Various ongoing and completed genome projects at the Ensembl webpage (http://www.ensembl.org/index.html) were also searched (based on human integrin sequences and on key words like “integrin”, “integrin alpha” or “integrin-like”) in order to finalize and create a dataset for our analysis (see Table 1 for list of organism and genomes utilized). In addition to the genome assembly searches, we also utilized the tBLASTn [52] to identify any fragments or Expression Sequences Tags (EST's) from organisms that diverged between the appearance of the Ascidians and Osteichthyes (bony fish). These specific searches included the genomes of the green spotted puffer [63], Nile tilapia [64], zebrafish [65], sea lamprey [41] and elephant shark [39]. All identified sequences were also cross-referenced against the conserved domain database (CDD; [18]) and the protein families database (PFAM; [66]) for confirmation.

### Sequence Alignment, Phylogenetic Tree Construction and Multivariate Analysis

Sequence alignments were carried out using TCOFFEE [67] and CLUSTALW [68] and examined for obvious errors. Phylogenetic trees were constructed using the Maximum Likelihood (ML) and Neighbor Joining (NJ) implementations in MEGA [69] and Phylip [70]. For the NJ trees, the Jones-Taylor-Thornton (JTT) distance matrix [43] was implemented for each set of alignments. Additionally, for the ML trees, the best-fit evolutionary model for the dataset was assessed using ProtTest [71] and MEGA; both programs reached the same conclusion and suggested the Whelan and Goldman (WAG) [44] substitution matrix with frequency model and gamma distribution with invariant sites (i.e. WAG+I+G+F) to be the best model to explain this dataset based on the Bayesian Information Criteria. Therefore, the WAG substitution matrix was implemented in order to derive the ML trees. For both the NJ and ML trees, the stability of the topology was explored using Felsenstein's bootstrap replication method [72] with 1000 bootstrap replicates. The ML and NJ trees were drawn with MEGA.

Bayesian phylogenetic analysis was performed using MrBayes [73] by implementing the Whelan and Goldman (WAG+I+G+F) model. Monte Carlo Markov Chain analysis was performed for 106 generations with a sampling frequency of 100 generations. The run was halted when the standard deviation of the split frequencies dropped below 0.01. The LnL graph (Log likelihood versus generation plot) was inspected and found to be satisfactory as there was no increasing or decreasing trend observed in the graph. Confidence level for the nodes was assessed with Bayesian posterior probabilities and the consensus tree was redrawn using Treegraph [74].

To complement the clusterings made by the three tree programs, we also supplied the distance data to a C-program program for multivariate analysis (PCA, MS Johnson). The program displays coordinates for each sequence and their locations such that the variance among the data is a maximum, and projections for various numbers of dimensions are possible. The three most informative dimensions, as a pseudo-PDB coordinate file, were visualized using Bodil [75].

### Structure Modeling and Identification of Functionally Important Residues

Protein structures were obtained from the Protein Data Bank [10]. The 2.1 Å resolution X-ray structure of the human integrin α2I domain in complex with the GFOGER tripeptide (IDZI; [15]) was used to model the structures of Pma_f1, Pma_f2 and Pma_f3. Structures of human αI domains were aligned using Vertaa in Bodil [75] and used as the basis to optimize the sequence alignments (optimal placement of gaps based on key functional residues and secondary structure) made using Malign [76]. Models were constructed using the Homodge package in Bodil and using Modeller [77] in Discovery studio (http://accelrys.com/products/discovery-studio/). Furthermore, energy minimization was included by using the Charmm force field [78] in Discovery studio.

Bodil [75] was used to visualize the model structures, explore the side-chain conformations using the rotamer utility, and to construct figures from the models. A simple C program, Surf2, was written to identify interactions between the α2I domain and the GFOGER tripeptide and structural water molecules (PDB code: 1DZI), and between the α1I domain NMR structure and GLOGEN tripeptide (PDB code: 3M32) and apo-form of the α1I domain (PDB code: 1PT6; [79]). A 4.2 Å distance cutoff between atoms was used to identify a contact. All contacts were confirmed visually using Bodil.

### Cloning and Protein Expression

Lamprey nucleic acid sequences for the predicted αI domain regions were synthesized by Eurofins MWG Operon (Germany) for Pma_f1, Pma_f2, and Pma_f3 and the genes were transferred into the pGEX-2T vector for expression. The expression strain *E. coli* BL21 tuner (Invitrogen, USA) was used for protein production, which was performed as earlier [58]. Human α2I domains (α2I wt and α2I E318W) of α2β1 integrin as well as glutathione S-transferase (GST) were expressed as described earlier [58]. Lamprey αI domains were expressed as either the full sequences below or the sequence minus the N-terminal amino acids that are highlighted in bold.

Pma_f1

**SGFNVSESYAPTLQ**KCGSYMDIVFVLDGSNSIYPWSDVQNFLVKTLQSFHIGPDQTQDDVCLPGANVVVVFKLSDTPLYERWGVSLVVLWRRWGMERGNDLNVYPSRSEAFSPERGARPDAQKVMIVVTDGESHDKYLLPEVIDQCERDGITRYAIAVLRSYSSNADDVARLINEVRSIASHPVERHFFNVTSEATLIDIVGTLGERIFSLEGTR

Pma_f2

**ADFQVTSTLTPAAQ**RCGLFMDIVIVLDGSNSIYPWQEVQNFVINIVKKFHIGPGQSRNGGGSTRFGVRTIHWHLGIARWACEGVQDVENIYSRPFVRTASALCQSLQVVRSEAFSPLFGAREGASKVMIVVTDGESHDSEDLTEAIAACERDNITRYAIAVLGYYKRKNIDPSNFISELKAISSEPEEKHFINVADEAALNDIVGTLGERIFSLEGTV

Pma_f3

**PNFQQLGSPFAPTMT**GCRSFLDIVLVLDGSNSIWPWPSVLDFLSSILETFSIGPGQTQVGIMQYGETVSNEMNLNQFTNKAQLKIAASKIPQRGGKVTNTAMGIEAARFFFFENGGRAEASKVMIVVTDGESSDAYKLPGVIKDCNDDGITRFGIAVLDYYISSNMNVEKLQAEIRSIASTPTEKYYFDVKSTGALVDITKALGERIYSLEGTS

For both the short and long versions of Pma_f1 αI and Pma_f3 αI we did not see any differences in their binding properties (not shown), however the short version of Pma_f2 αI was not expressible.

### Binding Studies

The following collagens were used in experiments: rat tail collagen I (Sigma Aldrich, USA), bovine collagen II (Chemicon, USA), mouse collagen IV (EHS mouse tumor; Becton-Dickinson, USA), and recombinant human collagen IX (a kind gift from Dr. Leena Ala-Kokko, University of Oulu). The GFOGER tripeptide was synthesized by Auspep (Australia). The triple-helical nature of the peptide has been checked with CD-spectroscopy.

Binding studies were performed as earlier [54]. In general, 96-well plates were coated with collagen (16.4 µg/ml) or GFOGER-peptide (5 µg/ml) or BSA (negative control; 1∶1 with Diluent II, Perkin-Elmer, USA) overnight at 4°C. Wells were washed once with PBS +2 mM MgCl2 and blocked with 1∶1 BSA-Diluent II, incubated for one hour at RT. Wells were washed once with PBS +2 mM MgCl2 and samples (all αI domains were used as a GST-fusion protein) were added to the wells for one hour at RT. Wells were then washed three times with PBS +2 mM MgCl2 and for each well Europium-labeled anti-GST antibody (Perkin Elmer, USA) was added in the Assay buffer (Perkin Elmer, USA) with 2 mM MgCl2. Wells were washed three times with PBS +2 mM MgCl2 and Enhancement Solution (Perkin Elmer, USA) was added to each well. Wells were measured using a Victor3-multilabel counter (Perkin Elmer, USA) using time-resolved fluorescence. Binding affinities of αI domains to rat collagen I were estimated by fitting the binding data using a hyperbolic function, which is identical to Hill's equation when h = 1.

## Supporting Information

File S1 Table S1: Sequences utilized in the phylogenetic analysis. Table S2. Residues in the α1I domain structure within 4.2 Å (non-hydrogen atoms) of the bound GLOGEN tripeptide (NMR structure; [21]) and equivalent residues in the human αI domains and the sequence fragments from the lamprey and hagfish. Where available, the sequence numbering is from a three-dimensional structure (PDB codes and resolution are indicated for the known X-ray structures). The metal ion at MIDAS is covalently bound to the tripeptide ligand. Residues from MIDAS (S13, S15, T81 and D114 in α1I, 3M32) are in italics and one residue, D11 in α1I (not listed) is absolutely conserved across all of the sequences. In the X-ray structure of α1I (PDB code: 1PT6; [79]) and this residue (D150 in 1PT6) binds to the metal at MIDAS via an intervening water molecule (WAT603). Table S3. Residues in the αLI domain structure within 4.2 Å (non-hydrogen atoms) of the bound ICAM and equivalent residues in the human αI domains and the sequence fragments from the lamprey and hagfish. Where available, the sequence numbering is from a three-dimensional structure (PDB codes and resolution are indicated for the known X-ray structures). The metal ion at MIDAS is covalently bound to the tripeptide ligand. Residues from MIDAS (S139, S141 and T206 in αLI, 1T0P) are in italics and two residues, D137 and D239 in αLI (not listed), are conserved across all of the sequences and functions to bind the metal at MIDAS via a water molecule (WAT943). Figure S1. Phylogenetic analysis of integrin sequences with the Bayesian method using MrBayes and based on the species and sequences listed in Tables 1 and S1. (A) Full-length sequence alignment of integrin α subunits his dataset contains the nearly full-length integrin α subunit from the sea lamprey Pma_f3 (highlighted in bold). (B) Tree based on the aligned common sequence region in all three lamprey sequence fragments Pma_f1, Pma_f2 and Pma_f3 (highlighted in bold). (C) Tree based on the alignment of the integrin αI domain sequences; this dataset includes the three lamprey αI domain sequences Pma_f1, Pma_f2 and Pma_f3 (highlighted in bold) and the hagfish fragment Ebu_f (highlighted in bold). Bayesian phylogenetic trees were constructed by implementing the Whelan and Goldman substitution matrix with frequency model and gamma distribution with invariant sites (WAG+I+G+F). Statistical support, in the form of the percentage posterior probability, was obtained with a MCMC run of 106 generations and the resulting percentage support value is indicated at each node. Figure S2. Phylogenetic analysis of integrin sequences with the Neighbor joining method using MEGA and based on the species and sequences listed in Tables 1 and S1. (A) Full-length sequence alignment of integrin α subunits his dataset contains the nearly full-length integrin α subunit from the sea lamprey Pma_f3 (highlighted in bold). (B) Tree based on the aligned common sequence region in all three lamprey sequence fragments Pma_f1, Pma_f2 and Pma_f3 (highlighted in bold). (C) Tree based on the alignment of the integrin αI domain sequences; this dataset includes the three lamprey αI domain sequences Pma_f1, Pma_f2 and Pma_f3 (highlighted in bold) and the hagfish fragment Ebu_f (highlighted in bold). Neighbor joining trees were constructed by implementing the Jones and Thornton (JTT) matrix. Statistical support for each phylogenetic tree was obtained with 1000 bootstrap replicates and the percentage bootstrap support value is indicated at each node. Figure S3. SDS PAGE of Pma_f1-3, human wild-type α2I, GST and molecular weight standards (st). SDS PAGE was run according to manufacturer's instructions using the GE Healthcare PhastSystem (GE, USA) and 8-25% gradient gel. Protein samples were adjusted to 300 ng/ml and the sample size was 1 µl. The gel was stained with Coomassie Brilliant Blue.
(DOC)
