# Supplementary material for: Early Chordate Origin of the Vertebrate Integrin αI Domains
Source: PLoS One. 2014 Nov 19;9(11):e112064. doi: 10.1371/journal.pone.0112064 (PMC4237329; doi:10.1371/journal.pone.0112064)

**Supplementary Material**

Table S1: Sequences utilized in the phylogenetic analysis.

Table S2. Residues in the α_1_I domain structure within 4.2 Å (non-hydrogen atoms) of the bound GLOGEN tripeptide (NMR structure; [21]) and equivalent residues in the human αI domains and the sequence fragments from the lamprey and hagfish. Where available, the sequence numbering is from a three-dimensional structure (PDB codes and resolution are indicated for the known X-ray structures). The metal ion at MIDAS is covalently bound to the tripeptide ligand. Residues from MIDAS (S13, S15, T81 and D114 in α1I, 3M32) are in italics and one residue, D11 in α_1_I (not listed) is absolutely conserved across all of the sequences. In the X-ray structure of α_1_I (PDB code: 1PT6; [79]) and this residue (D150 in 1PT6) binds to the metal at MIDAS via an intervening water molecule (WAT603).

Table S3. Residues in the α_L_I domain structure within 4.2 Å (non-hydrogen atoms) of the bound ICAM and equivalent residues in the human αI domains and and the sequence fragments from the lamprey and hagfish. Where available, the sequence numbering is from a three-dimensional structure (PDB codes and resolution are indicated for the known X-ray structures). The metal ion at MIDAS is covalently bound to the tripeptide ligand. Residues from MIDAS (S139, S141 and T206 in αLI, 1T0P) are in italics and two residues, D137 and D239 in α_L_I (not listed), are conserved across all of the sequences and functions to bind the metal at MIDAS via a water molecule (WAT943).

Figure S1. Phylogenetic analysis of integrin sequences with the Bayesian method using MrBayes and based on the species and sequences listed in tables 1 and S1. (A) Full-length sequence alignment of integrin α subunits his dataset contains the nearly full-length integrin α subunit from the sea lamprey Pma_f3 (highlighted in bold). (B) Tree based on the aligned common sequence region in all three lamprey sequence fragments Pma_f1, Pma_f2 and Pma_f3 (highlighted in bold). (C) Tree based on the alignment of the integrin αI domain sequences; this dataset includes the three lamprey αI domain sequences Pma_f1, Pma_f2 and Pma_f3 (highlighted in bold) and the hagfish fragment Ebu_f (highlighted in bold). Bayesian phylogenetic trees were constructed by implementing the Whelan and Goldman substitution matrix with frequency model and gamma distribution with invariant sites (WAG+I+G+F). Statistical support, in the form of the percentage posterior probability, was obtained with a MCMC run of 10^6^ generations and the resulting percentage support value is indicated at each node.

Figure S2. Phylogenetic analysis of integrin sequences with the Neighbor joining method using MEGA and based on the species and sequences listed in tables 1 and S1. (A) Full-length sequence alignment of integrin α subunits his dataset contains the nearly full-length integrin α subunit from the sea lamprey Pma_f3 (highlighted in bold). (B) Tree based on the aligned common sequence region in all three lamprey sequence fragments Pma_f1, Pma_f2 and Pma_f3 (highlighted in bold). (C) Tree based on the alignment of the integrin αI domain sequences; this dataset includes the three lamprey αI domain sequences Pma_f1, Pma_f2 and Pma_f3 (highlighted in bold) and the hagfish fragment Ebu_f (highlighted in bold). Neighbor joining trees were constructed by implementing the Jones and Thornton (JTT) matrix. Statistical support for each phylogenetic tree was obtained with 1000 bootstrap replicates and the percentage bootstrap support value is indicated at each node.

Figure S3. SDS PAGE of Pma_f1-3, human wild-type α_2_I, GST and molecular weight standards (st). SDS PAGE was run according to manufacturer’s instructions using the GE Healthcare PhastSystem (GE, USA) and 8-25% gradient gel. Protein samples were adjusted to 300 ng/ml and the sample size was 1 μl. The gel was stained with Coomassie Brilliant Blue.

Table S1.

| Organism | *Scientific name* | Sequence accession Codes | Abbreviation | Phylum/Family |
| --- | --- | --- | --- | --- |
| Human | *H. sapiens* | O75578*, AAF21944.1^#^ | Hsa α10 | Chordata/Hominidae |
| Human | *H. sapiens* | Q9UKX5*, AAD51919.2^#^ | Hsa α11 | Chordata/Hominidae |
| Human | *H. sapiens* | P17301*, AAM34795.1^#^ | Hsa α2 | Chordata/Hominidae |
| Human | *H. sapiens* | P56199*, NP_852478.1^#^ | Hsa α1 | Chordata/Hominidae |
| Human | *H. sapiens* | Q13349*, AAF62875.1^#^ | Hsa αD | Chordata/Hominidae |
| Human | *H. sapiens* | P20702*, NP_000878.2^#^ | Hsa αX | Chordata/Hominidae |
| Human | *H. sapiens* | P20701*, AAZ38713.1^#^ | Hsa αL | Chordata/Hominidae |
| Human | *H. sapiens* | P11215*, AAA59491.1^#^ | Hsa αM | Chordata/Hominidae |
| Human | *H. sapiens* | P38570*, NP_002199.3^#^ | Hsa αE | Chordata/Hominidae |
| Chimpanzee | *P. troglodytes* | H2PZU1*, XP_003308326.1^#^ | Ptr α10 | Chordata/Hominidae |
| Chimpanzee | *P. troglodytes* | H2Q9P5*, XP_510503.3^#^ | Ptr α11 | Chordata/Hominidae |
| Chimpanzee | *P. troglodytes* | H2QQV6*, XP_526928.2^#^ | Ptr α2 | Chordata/Hominidae |
| Chimpanzee | *P. troglodytes* | H2QQV4 *, XP_517769.2^#^ | Ptr α1 | Chordata/Hominidae |
| Chimpanzee | *P. troglodytes* | H2QB02*, XP_003807546.1^#^ | Ptr αM | Chordata/Hominidae |
| Horse | *E. caballus* | F6Q1A0*, XP_001499491.1^#^ | Eca α10 | Chordata/Equidae |
| Horse | *E. caballus* | F6VWM7*, XP_001495918.1^#^ | Eca α11 | Chordata/Equidae |
| Horse | *E. caballus* | F6RHG7*, XP_001494668.2^#^ | Eca α2 | Chordata/Equidae |
| Horse | *E. caballus* | F6SHD8*, XP_001494710.2^#^ | Eca α1 | Chordata/Equidae |
| Horse | *E. caballus* | F6WV98*, XP_001502733.2^#^ | Eca αE | Chordata/Equidae |
| Mouse | *M. musculus* | E9Q6R1*, NP_001074522.1^#^ | Mmu α10 | Chordata/Muridae |
| Mouse | *M. musculus* | Q7TQC3*, NP_795896.4^#^ | Mmu α11 | Chordata/Muridae |
| Mouse | *M. musculus* | Q3V3R4*, NP_001028400.2^#^ | Mmu α1 | Chordata/Muridae |
| Mouse | *M. musculus* | Q62469*, NP_032422.2^#^ | Mmu α2 | Chordata/Muridae |
| Mouse | *M. musculus* | E9PXZ7*, NP_001025043.3^#^ | Mmu αD | Chordata/Muridae |
| Mouse | *M. musculus* | Q9QXH4*, NP_067309.1^#^ | Mmu αX | Chordata/Muridae |
| Mouse | *M. musculus* | B7ZN91*, AAI45096.1^#^ | Mmu αL | Chordata/Muridae |
| Mouse | *M. musculus* | P05555*, P05555.2^#^ | Mmu αM | Chordata/Muridae |
| Mouse | *M. musculus* | A2T0Z1*, ABD49099.1^#^ | Mmu αE | Chordata/Muridae |
| Chicken | *G. gallus* | E1C311*, XP_413930.2^#^ | Gga α11 | Chordata/Phasianidae |
| Chicken | *G. gallus* | O42094*, NP_990400.1^#^ | Gga α1 | Chordata/Phasianidae |
| Chicken | *G. gallus* | F1N8T2*, XP_003643030.2^#^ | Gga α2 | Chordata/Phasianidae |
| Frog | *X. tropicalis* | F6YUK3*, XP_002932992.1^#^ | Xtr α11 | Chordata/Pipidae |
| Frog | *X. tropicalis* | F7B2H7*, XP_002933090.1^#^ | Xtr α1 | Chordata/Pipidae |
| Frog | *X. tropicalis* | F6UY99*, XP_002938301.1^#^ | Xtr αE | Chordata/Pipidae |
| Green spotted puffer | *T. nigroviridis* | H3DQB1*, [ENSTNIG00000019500](http://www.ensembl.org/Tetraodon_nigroviridis/Gene/Summary?g=ENSTNIG00000019500)^ | Tni α10 | Chordata/Tetraodontidae |
| Green spotted puffer | *T. nigroviridis* | H3CVZ9*, [ENSTNIG00000009560](http://www.ensembl.org/Tetraodon_nigroviridis/Gene/Summary?g=ENSTNIG00000009560)^ | Tni α11 | Chordata/Tetraodontidae |
| Green spotted puffer | *T. nigroviridis* | H3CQ26*, [ENSTNIG00000007551](http://www.ensembl.org/Tetraodon_nigroviridis/Gene/Summary?g=ENSTNIG00000007551)^ | Tni α1 | Chordata/Tetraodontidae |
| Green spotted puffer | *T. nigroviridis* | H3DET1*, [ENSTNIG00000015938](http://www.ensembl.org/Tetraodon_nigroviridis/Gene/Summary?g=ENSTNIG00000015938)^ | Tni α2 | Chordata/Tetraodontidae |
| Green spotted puffer | *T. nigroviridis* | H3DCL8*, [ENSTNIG00000015204](http://www.ensembl.org/Tetraodon_nigroviridis/Gene/Summary?g=ENSTNIG00000015204)^ | Tni αM_Like | Chordata/Tetraodontidae |
| Nile Tilapia | *O. niloticus* | I3JGP2*, XP_005472696.1^#^, [ENSONIG00000006374](http://www.ensembl.org/Oreochromis_niloticus/Gene/Summary?g=ENSONIG00000006374)^ | Oni α10 | Chordata/Cichlidae |
| Nile Tilapia | *O. niloticus* | I3JE40*, XP_005455722.1^#^, [ENSONIG00000005664](http://www.ensembl.org/Oreochromis_niloticus/Gene/Summary?g=ENSONIG00000005664)^ | Oni α11 | Chordata/Cichlidae |
| Nile Tilapia | *O. niloticus* | I3JL01*, XP_005456287.1^#^, [ENSONIG00000007572](http://www.ensembl.org/Oreochromis_niloticus/Gene/Summary?g=ENSONIG00000007572)^ | Oni α1 | Chordata/Cichlidae |
| Nile Tilapia | *O. niloticus* | I3KA00*, XP_003451125.2^#^, [ENSONIG00000014276](http://www.ensembl.org/Oreochromis_niloticus/Gene/Summary?g=ENSONIG00000014276)^ | Oni α2 | Chordata/Cichlidae |
| Nile Tilapia | *O. niloticus* | I3J5F1*, XP_005470170.1^#^, [ENSONIG00000003258](http://www.ensembl.org/Oreochromis_niloticus/Gene/Summary?g=ENSONIG00000003258)^ | Oni αL | Chordata/Cichlidae |
| Nile Tilapia | *O. niloticus* | I3KW08*, XP_003438691.1^#^, ENSONIG00000020087^ | Oni αM-A_Like | Chordata/Cichlidae |
| Nile Tilapia | *O. niloticus* | I3JAY8*, XP_005448390.1^#^, ENSONIG00000004788^ | Oni αM-B_Like | Chordata/Cichlidae |
| Zebrafish | *D. rerio* | E9QHI5*, XP_003200204.1^#^, [ENSDARG00000002507](http://www.ensembl.org/Danio_rerio/Gene/Summary?g=ENSDARG00000002507)^ | Dre α10 | Chordata/Cyprinidae |
| Zebrafish | *D. rerio* | F1QL14*, [ENSDARG00000007950](http://www.ensembl.org/Danio_rerio/Gene/Summary?g=ENSDARG00000007950)^ | Dre α11-B | Chordata/Cyprinidae |
| Zebrafish | *D. rerio* | F1QEW0*, NP_001166098.1^#^, ENSDARP00000052388^ | Dre α11-A | Chordata/Cyprinidae |
| Zebrafish | *D. rerio* | E9QDD7*, [ENSDARG00000074316](http://www.ensembl.org/Danio_rerio/Gene/Summary?g=ENSDARG00000074316)^ | Dre α1 | Chordata/Cyprinidae |
| Zebrafish | *D. rerio* | F1QC06*, XP_003199352.2^#^, ENSDARG00000062974^ | Dre α2 | Chordata/Cyprinidae |
| Zebrafish | *D. rerio* | A1A5I3*, XP_005171120.1^#^, ENSDARG00000044670^ | Dre αL | Chordata/Cyprinidae |
| Zebrafish | *D. rerio* | F1QSJ9*, XP_005156064.1^#^, ENSDARG00000002956 | Dre αX_like | Chordata/Cyprinidae |
| Zebrafish | *D. rerio* | E7F925*, [ENSDARG00000057787](http://www.ensembl.org/Danio_rerio/Gene/Summary?g=ENSDARG00000057787)^ | Dre αE | Chordata/Cyprinidae |
| Elephant shark | *C. milii* | SINCAMG00000009828^ | Cmi α11 | Chordata/Callorhinchidae |
| Elephant shark | *C. milii* | SINCAMG00000011330^ | Cmi α2 | Chordata/Callorhinchidae |
| Elephant shark | *C. milii* | SINCAMG00000011354^ | Cmi α1 | Chordata/Callorhinchidae |
| Elephant shark | *C. milii* | SINCAMG00000012649^ | Cmi αE | Chordata/Callorhinchidae |
| Sea lamprey | *P. marinus* | S4RDQ7*, ENSPMAG00000003066^ | Pma_f1 | Chordata/Petromyzontidae |
| Sea lamprey | *P. marinus* | S4RSW0*, ENSPMAG00000007533^ | Pma_f2 | Chordata/Petromyzontidae |
| Sea lamprey | *P. marinus* | S4RF57*, ENSPMAG00000003473^ | Pma_f3 | Chordata/Petromyzontidae |
| Common carp | *C. carpio* | Q98TF1*, BAB39134.1^#^ | Cca αL-1 | Chordata/Cyprinidae |
| Common carp | *C. carpio* | Q98TF0*, BAB39135.1^#^ | Cca αL-2 | Chordata/Cyprinidae |
| Inshore hagish | *E. burgeri* | BJ655520.1^#^ | Ebu_f | Chordata/Myxinidae |
| Sea pineapple | *H. roretzi* | Q9BPQ8*, BAB21479.1 | Hro α1 | Chordata/Pyuridae |
| Vase tunicate | *C. intestinalis* | Ci0100131118^+^ | Cin α1 | Chordata/Cionidae |
| Vase tunicate | *C. intestinalis* | Ci0100149446^+^ | Cin α2 | Chordata/Cionidae |
| Vase tunicate | *C. intestinalis* | Ci0100130596^+^ | Cin α3 | Chordata/Cionidae |
| Vase tunicate | *C. intestinalis* | Ci0100130838^+^ | Cin α4 | Chordata/Cionidae |
| Vase tunicate | *C. intestinalis* | Ci0100152002^+^ | Cin α5 | Chordata/Cionidae |
| Vase tunicate | *C. intestinalis* | Ci0100131399^+^ | Cin α6 | Chordata/Cionidae |
| Vase tunicate | *C. intestinalis* | Ci0100152615^+^ | Cin α7 | Chordata/Cionidae |
| Vase tunicate | *C. intestinalis* | Ci0100130149^+^ | Cin α8 | Chordata/Cionidae |

#, derived from Genbank; *, derived from Uniprot; ^, derived from Ensembl; +, sequences from the ascidian *C. intestinalis* were obtained from the supplementary material of Ewan et al. [31]. It is important to note here that the sequence accession codes used by Ewan et al. [31] are no longer recognized by the JGI database services.

Table S2.

| α1I, 3M32 | *S13* | N14 | *S15* | Y17 | P18 | R79 | Q80 | *T81* | *D114* | G115 | E116 | H118 | R148 | F156 |
| --- | --- | --- | --- | --- | --- | --- | --- | --- | --- | --- | --- | --- | --- | --- |
| α_1_I, 1PT6, 1.87 Å | S152 | N153 | S154 | Y156 | P157 | R218 | Q219 | T220 | D253 | G254 | E255 | H257 | R287 | F295 |
| α_2_I | S153 | N154 | S155 | Y157 | P158 | D219 | L220 | T221 | D254 | G255 | E256 | H258 | R288 | L296 |
| α_10_I | S | N | S | Y | P | R | E | T | D | G | E | H | R | F |
| α_11_I | S | N | S | Y | P | T | E | T | D | G | E | H | R | F |
| Pma_f1 | S | N | S | Y | P | M | E | R† | D | G | E | H | S | L |
| Pma_f2 | S | N | S | Y | P | V | R | T | D | G | E | H | R | F |
| Pma_f3 | S | N | S | W | P | K | V | T | D | G | E | S | S | L |
| Ebu αf | S | R | S | T | D | * | G | T | D | G | E | D | ? | ? |
| α_L_I, 3F74, 1.70 Å | S139 | M140 | S141 | Q143 | P144 | * | L205 | T206 | D239 | G240 | E241 | T243 | * | S270 |
| α_M_I, 1IDO, 1.70 Å | S141 | G142 | S143 | I146 | P147 | * | R208 | T209 | D242 | G243 | E244 | F246 | * | S280 |
| α_X_I 1N3Y 1.65 Å | S140 | G141 | S142 | S144 | S145 | * | F206 | T207 | D240 | G241 | K242 | E244 | * | S278 |
| α_D_I | S | G | S | D | Q | * | L | T | D | G | Q | Y | * | A |
| α_E_I | S | G | S | D | P | * | V | T | D | G | Q | F | * | T |

*, no equivalent or aligned residue; ?, residue not present in the sequence fragment; †, alignment uncertain at the position - no threonine present nearby in the sequence and replacement of arginine with threonine did not alter binding to collagens of the expressed mutant (data not shown).

Table S3.

| αLI 1T0P 1.70 Å | *S139* | M140 | *S141* | Q143 | T175 | L203 | L204 | L205 | *T206* | N207 | E241 | A242 | T243 | D244 | K263 | H264 |
| --- | --- | --- | --- | --- | --- | --- | --- | --- | --- | --- | --- | --- | --- | --- | --- | --- |
| α_M_I 1IDO 1.70 Å | S142 | G143 | S144 | I146 | E178 | L206 | G207 | R208 | T209 | H210 | E244 | K245 | F246 | D248 | D273 | A274 |
| α_X_I 1N3Y 1.65 Å | S140 | G141 | S142 | S144 | N176 | Q204 | G205 | F206 | T207 | Y208 | K242 | K243 | E244 | D246 | L271 | A272 |
| α_D_I | S | G | S | D | N | K | G | L | T | F | Q | K | Y | D | H | A |
| α_E_I | S | G | S | D | G | G | S | V | T | K | G | I | F | D | E | E |
| Ebu αf | S | R | S | T | S | K | A | G | T | N | E | S | * | D | ? | ? |
| Pma_f1 | S | N | S | Y | A | W | G | E | R† | G | E | S | H | D | * | * |
| Pma_f2 | S | N | S | Y | V | P | F | R | T | A | E | S | H | D | * | * |
| Pma_f3 | S | N | S | W | E | G | G | V | T | N | E | S | S | D | * | * |
| α_1_I 1PT6 1.87 Å | S152 | N153 | S154 | Y156 | E188 | G216 | G217 | Q219 | T220 | M221 | E255 | S256 | H257 | D258 | * | * |
| α_2_I 1AOX 2.10 Å | S153 | N154 | S155 | Y157 | N189 | G217 | G218 | L220 | T221 | N222 | E256 | S257 | H258 | D259 | * | * |
| α_10_I | S | N | S | Y | E | E | G | E | T | K | E | S | H | D | * | * |
| α_11_I | S | N | S | Y | E | G | G | E | T | R | E | S | H | D | * | * |

*, no equivalent or aligned residue; ?, residue not present in the sequence fragment; †, alignment uncertain at the position - no threonine present nearby in the sequence and replacement of arginine with threonine did not alter binding to collagens of the expressed mutant (data not shown).

Figure S1.


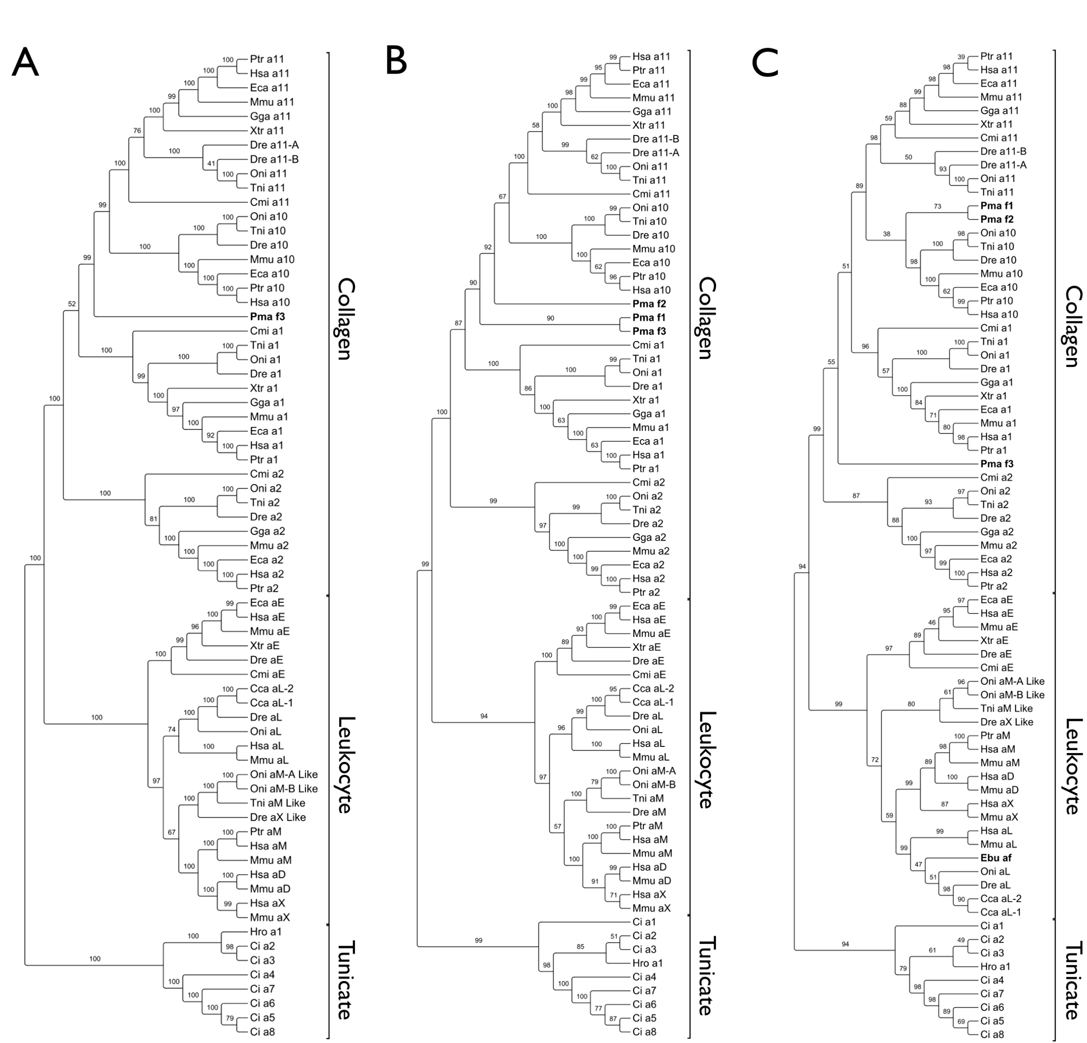


Figure S2.


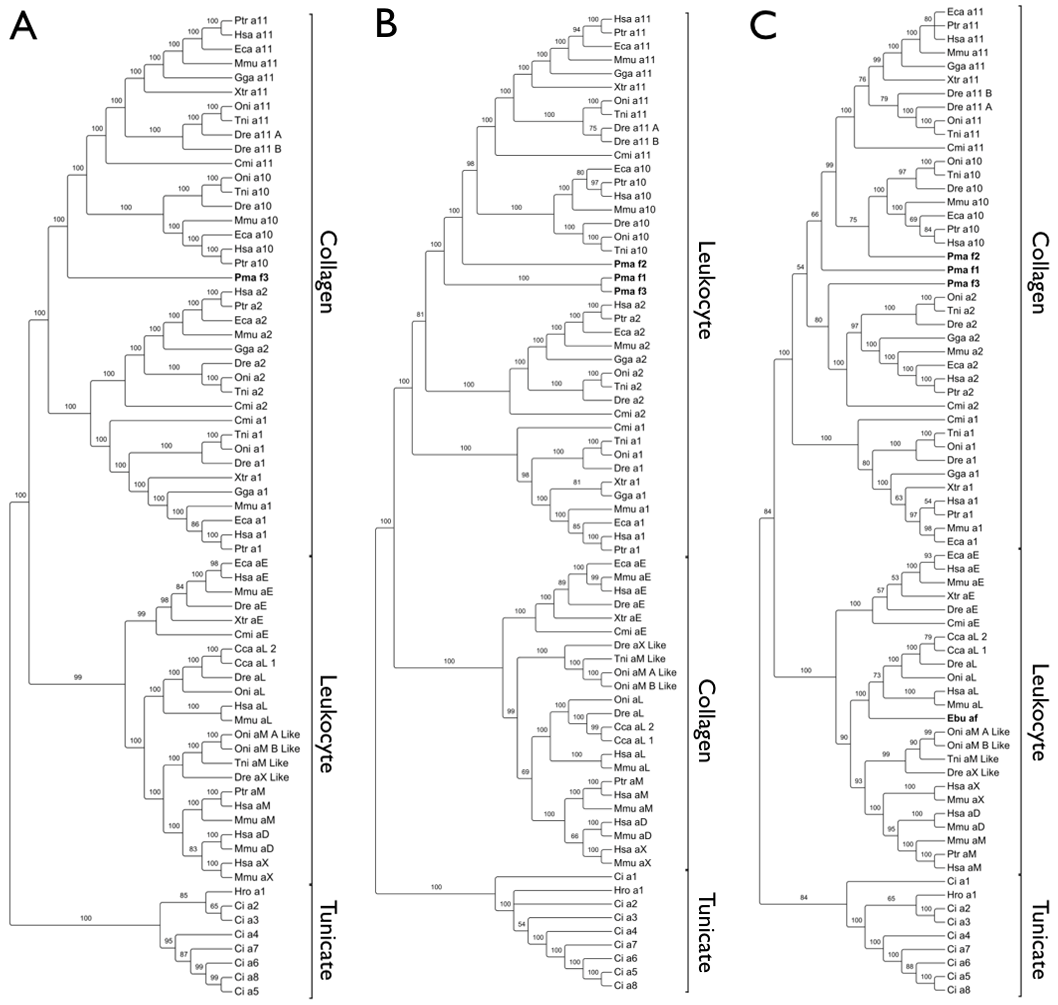


Figure S3.


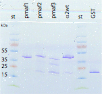

Supplement: File S1 — Table S1: Sequences utilized in the phylogenetic analysis. Table S2. Residues in the α1I domain structure within 4.2 Å (non-hydrogen atoms) of the bound GLOGEN tripeptide (NMR structure; [21]) and equivalent residues in the human αI domains and the sequence fragments from the lamprey and hagfish. Where available, the sequence numbering is from a three-dimensional structure (PDB codes and resolution are indicated for the known X-ray structures). The metal ion at MIDAS is covalently bound to the tripeptide ligand. Residues from MIDAS (S13, S15, T81 and D114 in α1I, 3M32) are in italics and one residue, D11 in α1I (not listed) is absolutely conserved across all of the sequences. In the X-ray structure of α1I (PDB code: 1PT6; [79]) and this residue (D150 in 1PT6) binds to the metal at MIDAS via an intervening water molecule (WAT603). Table S3. Residues in the αLI domain structure within 4.2 Å (non-hydrogen atoms) of the bound ICAM and equivalent residues in the human αI domains and the sequence fragments from the lamprey and hagfish. Where available, the sequence numbering is from a three-dimensional structure (PDB codes and resolution are indicated for the known X-ray structures). The metal ion at MIDAS is covalently bound to the tripeptide ligand. Residues from MIDAS (S139, S141 and T206 in αLI, 1T0P) are in italics and two residues, D137 and D239 in αLI (not listed), are conserved across all of the sequences and functions to bind the metal at MIDAS via a water molecule (WAT943). Figure S1. Phylogenetic analysis of integrin sequences with the Bayesian method using MrBayes and based on the species and sequences listed in Tables 1 and S1. (A) Full-length sequence alignment of integrin α subunits his dataset contains the nearly full-length integrin α subunit from the sea lamprey Pma_f3 (highlighted in bold). (B) Tree based on the aligned common sequence region in all three lamprey sequence fragments Pma_f1, Pma_f2 and Pma_f3 (highlighted in bold). (C) Tree based on the a [file pone.0112064.s001.doc]
